# Human-Induced Neurons from Presenilin 1 Mutant Patients Model Aspects of Alzheimer’s Disease Pathology

**DOI:** 10.3390/ijms21031030

**Published:** 2020-02-04

**Authors:** Sean Schrank, John McDaid, Clark A. Briggs, Sarah Mustaly-Kalimi, Deanna Brinks, Aiden Houcek, Oded Singer, Virginie Bottero, Robert A. Marr, Grace E. Stutzmann

**Affiliations:** 1 Center for Neurodegenerative Disease and Therapeutics, Rosalind Franklin University of Medicine and Science, North Chicago, IL 60064, USA; sean.schrank@rosalindfranklin.edu (S.S.); John.McDaid@rosalindfranklin.edu (J.M.); Clark.Briggs@rosalindfranklin.edu (C.A.B.); sarah.mustaly@rosalindfranklin.edu (S.M.-K.); Virginie.Bottero@rosalindfranklin.edu (V.B.); 2 School of Graduate and Postdoctoral Studies, Rosalind Franklin University of Medicine and Science, North Chicago, IL 60064, USA; 3 Chicago Medical School, Rosalind Franklin University of Medicine and Science, 3333 Green Bay Rd. North, Chicago, IL 60064, USA; Deanna.Brinks@rosalindfranklin.edu; 4 Lake Forest College, Lake Forest, IL 60045, USA; Aiden.Houcek@rosalindfranklin.edu; 5 Weizmann Institute of Science, Life Sciences Core Facilities, Rehovot 76100, Israel; oded.singer@weizmann.ac.il

**Keywords:** iPSC, Alzheimer’s disease, amyloid, tau, calcium, fibroblast, human, IL-18, ryanodine receptor

## Abstract

Traditional approaches to studying Alzheimer’s disease (AD) using mouse models and cell lines have advanced our understanding of AD pathogenesis. However, with the growing divide between model systems and clinical therapeutic outcomes, the limitations of these approaches are increasingly apparent. Thus, to generate more clinically relevant systems that capture pathological cascades within human neurons, we generated human-induced neurons (HiNs) from AD and non-AD individuals to model cell autonomous disease properties. We selected an AD patient population expressing mutations in presenilin 1 (mPS1), which is linked to increased amyloid production, tau pathology, and calcium signaling abnormalities, among other features. While these AD components are detailed in model systems, they have yet to be collectively identified in human neurons. Thus, we conducted molecular, immune-based, electrophysiological, and calcium imaging studies to establish patterns of cellular pathology in this patient population. We found that mPS1 HiNs generate increased Aβ_42_ and hyperphosphorylated tau species relative to non-AD controls, and exaggerated ER calcium responses that are normalized with ryanodine receptor (RyR) negative allosteric modulators. The inflammasome product, interleukin-18 (IL-18), also increased PS1 expression. This work highlights the potential for HiNs to model AD pathology and validates their role in defining cellular pathogenesis and their utility for therapeutic screening.

## 1. Introduction

### 1.1. PS1 Mutant Human-Induced Neurons Model Aspects of AD Pathology

Alzheimer’s disease (AD) is the leading cause of dementia in aged people [1], and as this demographic grows, AD represents a looming financial and socioeconomic threat with little success in current treatment options. Despite significant resource investment in AD research, a disease-modifying treatment has yet to be found despite promising outcomes in AD animal models. This dichotomy, in large part, likely reflects a limitation in the available model systems which rely heavily on exogenous (over)expression of human AD mutations in rodents or in non-excitable cell lines. While much information has been obtained from these models, the clear disconnect between successful therapeutic indicators in model systems and failed human clinical trials indicates a significant gap in translation. Thus, the ability to generate human AD neuronal cells may provide much-needed insight into disease mechanisms and therapeutic targets that are currently unobtainable from animal models or cell lines.

### 1.2. PS1 Mutations and AD Pathology

The genetic analysis of familial AD (FAD) patients led to the discovery that mutations in either presenilin-1 (PS1), presenilin-2 (PS2), or the amyloid precursor protein (APP) cause FAD [2,3,4,5]. Compared to sporadic AD, FAD is a more aggressive form resulting in earlier disease onset and more rapid symptom development [6]. PS1 mutations result in increased Aβ_42_ production and thus senile plaque formation, as well as earlier age of onset and more aggressive disease severity. These observations were used to support the amyloid cascade hypothesis which postulates that Aβ causes AD [7], and thus clearing Aβ plaques should halt memory decline in AD. However, after multiple phase III clinical trial failures, it is evident that the cause and driving mechanisms of AD are poorly understood, and further exploration of the link between presenilin mutations and AD pathology is required.

One of the more well-defined functions of PS1, as part of the gamma secretase complex, is to cleave APP and generate soluble Aβ peptides, of which Aβ_42_ is considered the most pathogenic. The toxicity of Aβ peptides may be mediated through interference of signal transduction pathways such as Erk/MAPK, CaMKII, phosphatidylinositol 3-kinase-activated protein Akt/protein kinase B(PI_3_KB), TOM1, and others [8,9,10], along with potentiation of NMDA receptor currents [11], enhanced ryanodine receptor subtype 2 (RyR2) protein and message expression [12,13] and formation of calcium-permeable ionophores [14], all of which result in diminished neuronal function. The presence of histopathological hallmarks of Aβ and of pathological tau species indicates a probable diagnosis of AD post-mortem [15,16,17,18]. Tau functions as a microtubule-stabilizing protein that contributes to cytoskeletal stability [19]. In AD, tau becomes hyper-phosphorylated and aggregates into paired helical filaments (PHFs), which destabilize the neuronal cytoskeleton and interfere with axonal transport and neuronal signaling with eventual neurotoxicity [20].

Further investigation of FAD mutations revealed the link of mutant presenilin to calcium dyshomeostasis [21,22,23]. Calcium is a ubiquitous signaling ion that is fundamental to neuronal health and function including synaptic plasticity and memory encoding; as such, interference with calcium regulation at any level can have deleterious effects [24]. FAD mutations are associated with early dysregulation of intracellular calcium stores, such as the ER through IP_3_Rs and RyRs, prior to the emergence of senile plaques and NFTs [25,26,27]. Furthermore, aberrant increases in RyR-mediated calcium release contributes to synaptic decay in AD, and normalization of RyR-calcium responses restores synaptic structure and function, with an associated reduction of amyloid load [28]. Furthermore, PS1 is linked to RyR expression and function, and PS knockout mice demonstrate reduced neuronal RyR expression, diminished RyR calcium release, and impairments in synaptic plasticity [29]. To date, pathogenic defects in ER calcium signaling have been characterized in several AD mouse models, cellular models, and non-excitable human cells [25,26,27,28] but have yet to be validated in neurons from AD patients.

With the identification of several immune-related risk factor polymorphisms in genes such as TREM2, CD33, HLA-DRB5, MS4A6A, and ABCA7, it is clear that the immune response plays a role in AD pathogenesis [30]. Research dating back 20 years has demonstrated the involvement of pro-inflammatory cytokine IL-1β in AD pathogenesis, with effects ranging from activation of microglia and astrocytes to inhibition of LTP [31,32,33,34,35]. Recent work has implicated the involvement of the NLRP3 inflammasome in AD pathogenesis [36], suggesting that both IL-1β and IL-18 are intimately linked to AD pathogenesis. IL-18 stimulation of the neuron-like SH-SY5Y cell line increased expression of APP and APP processing enzymes such as BACE-1 and PS1 [37], further suggesting that IL-18 signaling may drive aspects of AD pathology.

The ability to model these convergent phenotypes of AD is crucial to understanding disease pathogenesis, and here we seek to understand how mutant PS1-derived human-induced neurons (HiN) model AD pathology. We generated HiNs by converting AD patient fibroblasts into induced pluripotent stem cells (iPSC) and converting the iPSCs to HiNs by lentiviral vectors containing neurogenin-2 (NGN2) [38]. Using this method, we report robust conversion from iPSCs to HiNs that express mature neuronal protein markers and are electrophysiologically and synaptically active. This technique allows for the modeling of neurodegenerative disease mechanisms in living AD human neurons, which represents a significant advancement and critical turning point for the field. Here we demonstrate within mutant (A246E and M146L) PS1-expressing human neuronal networks, the confluence of complex AD pathologies including Aβ, tau hyper-phosphorylation, calcium dyshomeostasis, and neuroinflammation, thus providing a clinically relevant platform with which to identify effective therapeutic targets.

## 2. Results

### 2.1. Generation of Human-Induced Neurons

**Figure 1:**
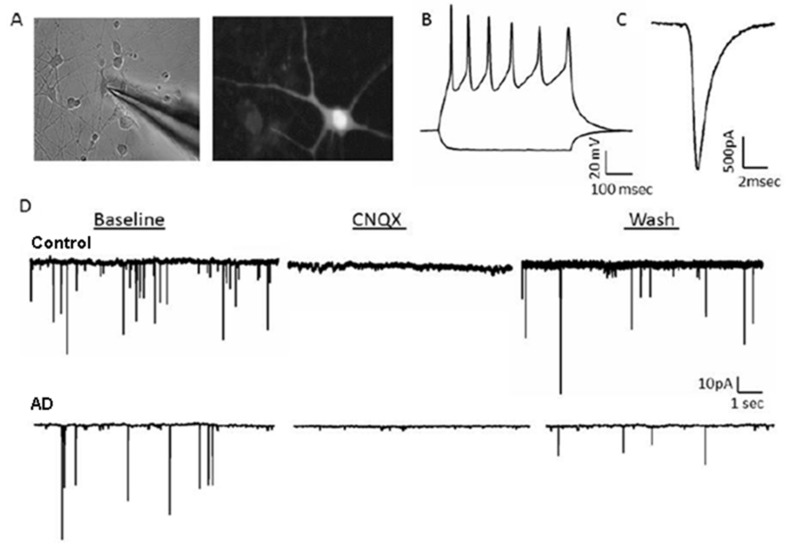
Human-induced neurons (HiNs) display spiking activity, mature Na+ currents, and spontaneous synaptic activity. (**A**) Left, representative IR-DIC image using an air 10× objective of mature HiNs with whole-cell patch clamp recording. Right, Fura-2 filled neuron imaged using 2-photon fluorescence microscopy with a 40× water immersion objective. (**B**) Voltage-gated spiking activity in response to depolarizing current injection as assessed using whole-cell patch clamp electrophysiology. (**C**) Evoked Na+ currents in response to depolarizing current injection as assessed using whole-cell patch clamp electrophysiology. (**D**) Spontaneous glutamatergic excitatory postsynaptic currents (EPSCs) from both Alzheimer’s disease (AD) and Non-AD HiNs were reversibly blocked by the AMPA receptor antagonist CNQX.

Animal models expressing PS1 mutations have significantly advanced our understanding of AD pathology but have yet to lead to an effective therapy. The expanding field of cellular reprogramming has granted the ability to generate human neuronal tissue from non-neuronal cells, permitting the study of AD mechanisms in living human neurons for the first time. To identify the contribution of PS1 mutations to AD pathology in human neurons, we characterized 10 patient-derived iPSC clones using fibroblasts from six human patients (3 control and 3 mutant PS1) with each clone used to derive HiNs (Table S1). The resultant HiNs expressed mature neuronal markers (Table S1), demonstrating successful conversion from an iPSC to a neuronal state. Most importantly, HiNs were found to be electrophysiologically active (Figure 1), generating mature voltage-dependent action potentials (Figure 1B) and Na+ currents (Figure 1C), and spontaneous and evoked synaptic activity that was reversibly blocked by the AMPA receptor antagonist CNQX (Figure 1D). The successful generation of physiologically active neurons indicates this conversion methodology is an effective approach to generate human neuronal tissue for examining AD pathogenesis in a relevant species cell type.

### 2.2. Human-Induced Neurons Model Histopathological Hallmarks of AD

**Figure 2:**
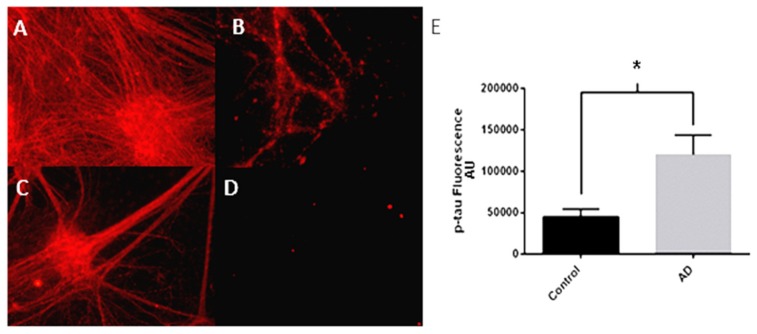
Hyperphosphorylated tau levels are increased in AD HiN. Fixed HiN cultures were assayed for phosphorylated tau by immunocytochemistry against hyperphosphorylated tau (Ser202, Thr 205). Representative cultures of AD HiNs display robust staining (**A**,**B**,**C**), while HiNs from non-AD patients show minimal labeling (**D**) as measured by widefield fluorescence microscopy using a 20× air objective. (**E**) Quantitation of mean fluorescence intensity comparing ROI averaged data from non-AD control (black bar, *n* = 16) and AD HiN (gray bar, *n* = 16). * = *p* < 0.01.

**Figure 3:**
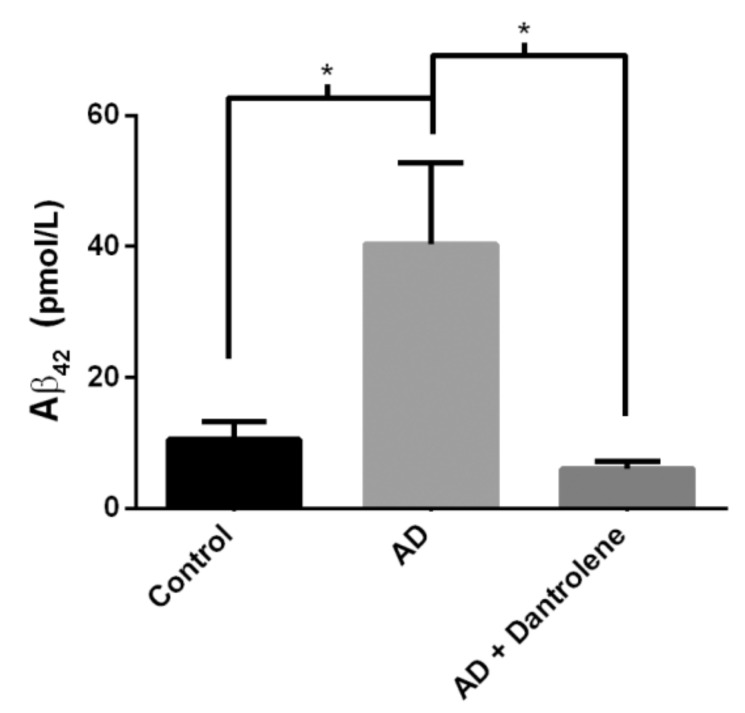
Dantrolene treatment reduces Aβ_42_ production. HiNs at maturity were treated overnight with 10 µM dantrolene or vehicle after complete media change. Supernatants were collected and assayed by specific ELISA. AD HiNs (*n* = 7 wells) produce significantly more Aβ_42_ than WT HiNs (*n* = 8 wells). Additionally, 10 µM dantrolene treatment significantly reduced Aβ_42_ production to that of non-AD control levels (*n* = 4 wells,). *= *p* < 0.05.

To evaluate the effects of PS1 mutations on human AD, we assessed HiNs for both hyper-phosphorylated tau and Aβ_42_ production. Three weeks post-neuronal induction, HiNs were fixed and immunostained for hyperphosphorylated tau using a phospho-specific antibody (clone identity AT8). We identified that AD HiNs (*n* = 16 wells) have elevated tau phosphorylation compared to non-AD HiNs (*n* = 16 wells) (two-tailed t-test, t _(1,30)_ = 2.93; *p* < 0.01. Figure 2). HiN culture media was also assayed for the presence of Aβ_42_ by specific ELISA, and it was determined that the AD HiNs produced significantly more Aβ_42_ than non-AD control HiN. Notably, this increase in a pathogenic amyloid species was ameliorated upon incubation with dantrolene, implicating intracellular calcium dyshomeostasis via the RyR as a significant contributing factor in the generation of Aβ_42_ (One-way ANOVA, F_(2,18)_ = 5.058; *p* < 0.05. Figure 3). This demonstrates that the human PS1 mutation is sufficient for both enhanced Aβ_42_ production and increased tau-hyperphosphorylation in neurons and supports the use of this model system for studying FAD pathogenesis.

### 2.3. Human-Induced Neurons from AD Patients Exhibit Exaggerated ER Calcium Release

**Figure 4:**
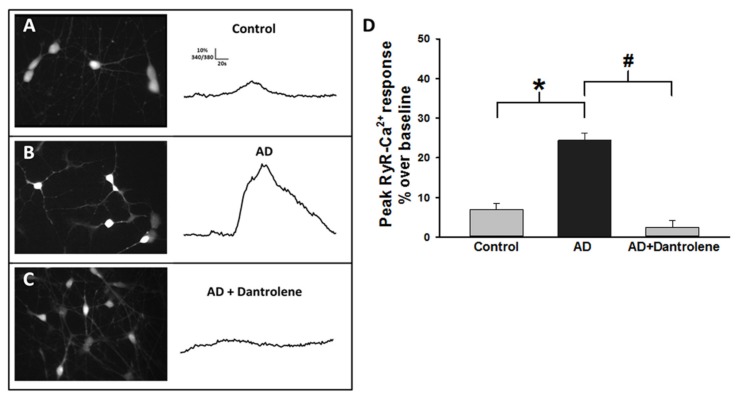
Ryanodine receptor (RyR)-evoked calcium release is greater in AD HiNs and normalized by dantrolene. (**A**–**C**) RyR-evoked calcium release (via bath application of 10 mM caffeine) was measured using the ratiometric indicator Fura-2AM from non-AD HiNs (top), AD HiNs (middle), and AD HiNs treated with the RyR negative allosteric modulator, dantrolene (10 µM; bottom). Endoplasmic reticulum (ER) calcium release was measured as peak change in 340/380 ratio over baseline. Representative traces of non-AD control, AD, and AD + dantrolene treated HiNs are displayed in A–C on right. (**D**) Averaged peak RyR-evoked calcium responses from Non-AD, AD, and AD+dantrolene treated HiNs. *#*p* < 0.05.

Investigation into upstream pathogenic mechanisms of AD has revealed that ER-calcium dysregulation occurs prior to the generation of pathological Aβ species and tau hyper-phosphorylation [28,39,40,41]. We measured RyR-evoked calcium release in PS1 mutant HiNs, and found that the AD PS1 HiNs have exaggerated RyR calcium responses compared to cognitively normal controls (Data representative of all HiNs sampled (Non-AD *n*=125 ([Clone 17:*n* = 17] [Clone 37:*n* = 13] [Clone 38:*n* = 49] [Clone 46:*n* = 20] [ Clone 67:*n* = 25]) AD=189([ Clone 11:*n* = 28][ Clone 25:*n* = 74][ Clone 26:*n* = 24][ Clone 49:*n* = 16][ Clone 60:*n* = 47]). Mature HiNs derived from three mutant PS1 and three non-mutant PS1 individuals were assessed for peak RyR-evoked calcium release (with 10 mM caffeine) using the fluorescent ratiometric calcium indicator Fura-2AM (5 μM). We found that AD HiNs have a significantly larger somatic RyR-calcium response (24.4% ± 1.8% over baseline; *n* = 189) than non-AD HiNs (7.8% ± 1.4% over baseline; *n* = 125; F_(2,318)_ = 24.38; *p* < 0.001), effects that were mitigated with dantrolene treatment in the AD neurons (2.5% ± 1.7% over baseline; *n* = 5; Figure 4A). This is consistent with similar studies in AD mouse models which demonstrated normalized ER calcium release, reduced amyloid and plaque generation, and restored synaptic structure after dantrolene treatment [28,42]. The exaggerated calcium response is normalized by incubation with the RyR negative allosteric modulator, dantrolene (10 µM for 20 min; Figure 4C,D), suggesting that RyR is predominantly underlying the exaggerated ER calcium response in human AD neurons. This demonstrates for the first time a functional relationship between AD-associated human PS1 mutations and dysregulated RyR function in human neurons; this is particularly relevant in light of the central role intracellular calcium signaling plays in synaptic transmission, plasticity, and memory encoding, as well as oxidative stress, apoptosis, and inflammation, [39,43,44]. Notably, these findings in HiN align closely with detailed studies in multiple AD mouse models that demonstrate that altered upstream ER calcium homeostasis drives synaptic loss, pathogenic amyloid and tau formation, and propagation of inflammatory responses [28,29,43,44].

### 2.4. Normalizing RyR-Evoked Calcium Release Reduces Aβ_42_ Production in FAD HiNs

As dantrolene mitigated the increased RyR calcium response in AD HiNs, we next tested if dantrolene treatment could reduce Aβ_42_ production as had previously been demonstrated in transgenic mouse models [28,45]. Mature HiNs generated from AD patients and cognitively normal non-AD controls were treated with 10 µM dantrolene overnight, then supernatants collected for analysis. Using an Aβ_42_-specific ELISA, we determined that 10 µM dantrolene treatment significantly reduced Aβ_42_ production to that of WT levels (Figure 3). This demonstrates that dantrolene treatment has the ability to both normalize RyR calcium signaling abnormalities and reduce pathogenic Aβ_42_ production providing evidence that targeting intracellular calcium channels may be a viable candidate for AD treatment.

### 2.5. IL-18 Increases PS1 Expression in Human-Induced Neurons

**Figure 5:**
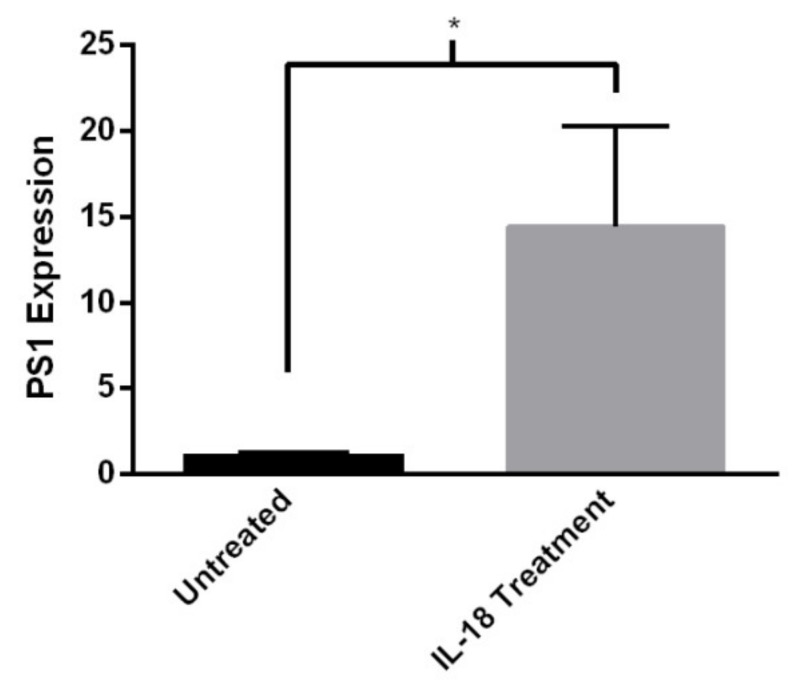
IL-18 alters PS1 gene expression in HiNs. Four HiN lines (non-AD control and AD) were stimulated with 150 ng/mL of IL-18 for 6 h, and SYBR based qRT-PCR of extracted RNA was subsequently performed. Results demonstrate an upregulation of PS1 message in response to IL-18 incubation. PS1 expression is displayed relative to tubulin. (untreated *n* = 12, IL-18 treatment *n* = 12. *n* = 1qRT-PCR replicate) *=*p* < 0.05.

Of growing interest to the AD community is the contribution of inflammation and inflammatory processes to disease pathology. It is appreciated that AD presents with inflammation including microglia and astrocytic production of the pro-inflammatory cytokines IL-1β and IL-18 [36]. The effect of IL-18 on human neuroblastoma cells (SH-SY5Y) has previously been reported [37], in which IL-18 significantly upregulated the expression of several AD-related genes such as PS1 and BACE1. We analyzed HiNs exposed to IL-18 or vehicle and assessed the expression levels of PS1, BACE1, and RyR2 by quantitative real-time PCR of cDNA extracted from these cells. While there was no effect of IL-18 stimulation on BACE1 and RyR2 gene expression levels (data not shown, *p* > 0.05), it did affect HiN PS1 expression after 6 h of exposure (two-tailed t-test, t_(1,22)_ = 2.24; *p* < 0.05. Figure 5). This confirmatory finding suggests that pro-inflammatory cytokines have direct effects on neurons and can alter the expression of genes linked to AD.

## 3. Discussion

The ability to model AD in human excitable cells represents a significant advancement to the field. For the first time, we are able to directly examine the effect of AD-causing PS1 mutations on developing pathophysiology in human neurons. Rodent models have been instrumental in our initial exploration of AD, and the advent of reprogramming technology will further advance our understanding in more relevant cellular systems. We present here an analysis of our HiN culture system expressing human PS1 mutations. As expected, the PS1 A246E mutation produced significantly more Aβ_42_ than non-mutant PS1control HiNs. Based on analogous studies in a variety of model systems, this likely reflects enhanced gamma secretase activity which is a consequence of these PS1 mutations [46,47,48,49,50]. This consistent finding in the HiNs is an important indicator that the processes driving amyloid pathology can be modeled in human neurons.

The mutant PS1 HiNs also generate increased levels of tau hyperphosphorylation relative to non-AD controls. The link between PS1 mutations and tau hyperphosphorylation could be explained by upregulation of kinases implicated in tau pathology such as GSK3β or CDK5 [50,51], but whether or not this is an intrinsic characteristic of HiNs bearing PS1 mutations, or a product of the Aβ in the culture system remains to be seen. However, it is unlikely that Aβ would accumulate to sufficient levels to result in tau pathology in our culture system because the relative amount of extracellular space is much greater in cultured cells when compared to brain tissue. Thus, the effect on tau pathology is more likely a result of cell-autonomous signaling alterations due to the PS1 mutation. An alternative signaling mechanism may reflect calcium-mediated upregulation of the aforementioned tau kinases [26]. Future experiments dedicated to the exploration of tau pathology will address this.

Additionally, we validated that RyR-mediated calcium release is significantly increased in mutant PS1 HiN neurons, as has been observed in multiple AD mouse models and cell culture systems. Surveyed across 10 HiN clones, the mutant PS1 HiNs demonstrate significantly increased RyR-mediated calcium release relative to non-AD controls. PS1 mutations have long been associated with calcium dyshomeostasis in a variety of animal and cellular models prior to the emergence of plaques and tangles. First identified was ER-mediated calcium dysregulation via IP_3_R- and RyR-evoked calcium channels [21,23,39,52]. Cellular studies demonstrated that exogenous expression of mutant PS1 in oocyte models potentiated IP_3_R-evoked calcium responses relative to wild type PS1 [22]. Additionally, human non-neuronal cells obtained from pre-symptomatic FAD patients demonstrated enhanced calcium responses to IP_3_-generating stimuli relative to age-matched non-AD cohorts and non-FAD family members [39]. Following the identification of potentiated IP_3_R-evoked calcium responses in AD models, it was discovered that exaggerated RyR calcium signaling is present prior to the emergence of the traditional histopathological hallmarks and cognitive decline in AD [25,27,53]. This exaggerated RyR calcium response in AD neurons results in synaptic transmission deficits including reduced presynaptic neurotransmitter vesicle pools and increased after-hyperpolarization potentials mediated by Ca^2+^-activated K^+^ channels both of which contribute to synaptic decline and loss [38,54,55]. This current research demonstrates that PS1 mutations contribute to RyR-mediated calcium dysregulation in human neurons from AD patients. We also found that normalizing RyR function with dantrolene reduced the production of Aβ_42_, again supporting the role of RyR in AD as well as the utility of HiNs for modeling disease.

Lastly, we have demonstrated the functionality of the HiN system to model aspects of neuro-inflammation. Several experiments have demonstrated the importance of the immune response in AD pathology development. The development of NLRP3 -/- x APP/PS1 transgenic rodent models demonstrates that by reducing inflammation in AD models, histopathology hallmarks and cognitive decline can be mitigated [36]. NLRP3 inflammasome activation leads to the production of mature IL-1β and IL-18. Both IL-1β and IL-18 have been implicated not only in the inflammatory response in AD, but also to the upregulation of AD-related genes such as PS1, APP, and BACE1. We demonstrate here that treating HiN cultures with IL-18 alters PS1 expression 6 h post-stimulation. This is consistent with previous findings in neuronal-like SH-SY5Y cell lines [37]. Contrary to what was seen in the SH-SY5Y cells, we did not detect significant elevations of BACE1 or APP. This may reflect the level of maturity of the HiNs or represent a deviation from the more artificial cell lines compared to HiNs. Future experiments will assess IL-18 effects in older HiN cultures, and further validate our initial findings using additional clones.

While these new developments are exciting and represent a significant advance for the neurodegeneration field, there are limitations regarding the use of HiN. This remains an artificial in vitro system which cannot fully recapitulate the complex environment of an integrated living system. The advent of 3-D culture systems and organoids will help address some of these concerns, but these too come with caveats regarding high variability and non-standardized approaches across research platforms. These HiN are derived from embryonic stem cells converted from fibroblasts, and during these transformations back to a pluripotent stem cell, the cells may lose age-related features and epigenetic signatures. This is of particular concern when studying age-related diseases such as AD. Also, there is a high degree of inherent variability in human samples which is compounded by potential clonal variability. Our analyses started with the study of the highly penetrant PS1 mutations to mitigate some of the issues with genetic variability. Studies assessing sporadic AD would require greater sample sizes and would benefit by utilizing direct reprogramming from patient somatic cells into neurons, and ‘skipping over’ the reversion to a stem cell state. This would eliminate iPSC clonal variability and presumably produce cells that more closely recapitulate the “aged” quality of the patient.

## 4. Methods

### 4.1. iPSC and HiN Generation

To generate HiN, healthy control- and AD patient- fibroblasts obtained from the Coriell Institute (Tables S1 and S2) were converted into iPSCs, using standard techniques involving gene delivery of the Yamanaka factors by vector or RNA transfection (following the manufacturer’s instructions for the ReproRNA-OKSGM kit, StemCell Technologies, Vancouver, BC, Canada). Fibroblasts were transfected and cultured for 14–21 days in REPRO-TSR medium before iPSC colonies were visible. Colonies were then picked and evaluated for viability (homogeneity of stem cells, colony size, ability to form new colonies) and iPSC specific markers (expression of OCT4, Sox2) to ensure successful reprogramming. Once verified as iPSCs (Table S1), colonies were converted to HiN by lentiviral vectors containing the transcription factor NGN2. iNs were selected for lentiviral transduction by puromycin resistance, allowed to mature over 14–28 days in Neural Basal Media (ThermoFisher, Waltham, MA, USA) with doxycycline (for transgene activation), puromycin (for selection), brain-derived neurotrophic factor (BDNF) and neurotrophin 3 (NT3) (PeproTech, Rocky Hill, NJ, USA ) (as previously described in [38]). We have experimented with a variety of growth substrates ranging from MEFs, laminin, fibronectin, and Matrigel™ on both glass and plastic coverslips. This process led us to use a thin coat Matrigel method combined with plastic (Thermanox) coverslips for physiology experiments as it increases HiN viability most likely through improved attachment. Group and sample sizes were determined from overviews of the literature and from tests for statistical significance, and assays were conducted to confirm reproducibility and consistency within clones over time. Variability was also reduced by requiring neuronal signaling properties, such as ionic channel activation, mature action potentials, and synaptic responses, to be present in cells prior to inclusion in the study. Sex as a biological variable was not specifically considered in this study, although groups were matched for age and sex; future studies will be designed to address this appropriately.

### 4.2. qRT-PCR

Quantitative real time PCR was performed using the ViiA7 platform (Molecular Quantification Laboratory at Rosalind Franklin University of Medicine and Science), with primers for Tubulin (FWD: TCCAGATTGGCAATGCCTG, REV: GGCCATCGGGCTGGAT), OCT4 (FWD: CCCCAGGGGCCCCATTTTCGTACC, REV:GGCACAAACTCCAGGTTTTC) SOX2 (FWD: ACACTGCCCCTCTCACCACAT, REV: GGGTTTTCTCCATGCTGTTTCT), NeuN (FWD: GTAGAGGGACGGAAAATTGAGG, REV: CATAGAATTCAGGCCCGTAGAC), MAP2 (FWD:CAGGAGACAGAGATGAGAATTCC, REV: CAGGAGTGATGGCAGTAGAC), RyR2 (FWD:GGCAGCCCAAGGGTATCTC, REV: ACACAGCGCCACCTTCATAAT, and PS1 (FWD: GACGACCCCAGGGTAACTC, REV: ACTGACTTAATGGTAGCCACGA). The delta-delta Ct method was used with Sybr Green (Evo Green, Midwestern Scientific, Valley Park, MO, USA) detection of amplicon products using standard amplification protocols. For IL-18 induction experiments, each HiN culture was assessed for gene expression by pooling triplicates from eight mature HiN lines in 24 wells (approximately 60,000 cells). qRT-PCR was performed in triplicate.

### 4.3. Immunohistochemistry

Mature HiNs were fixed overnight in 4% PFA, permeabilized with 1% Triton-X-100 for 5 min at room temperature, and then blocked using 6% normal goat serum at room temperature on a platform rocker for 30 min. HiNs were then incubated in primary antibody (hyperphosphorylated tau: AT8 antibody (ThermoFisher (MN1020) 1:1000), TRA-160 (Abcam (ab16288) 1:200), TRA-181 (Abcam (ab16289) 1:200) overnight at 4 °C on a platform rocker. Neurons were then rinsed 3x in 1xPBS, and incubated in secondary antibody (Alexafluor 594, ThermoFisher 1:1000) at room temperature on a platform rocker for two hours. Fluorescent images were then collected using a 20× air objective using a Nikon Eclipse TE 2000-S microscope (Melville, NY, USA) with an XCite series 120 PC illumination source, captured with Nikon Elements software (AR package, Melville, NY, USA). Data are presented as mean fluorescent intensity in randomly selected ROI in each representative image.

### 4.4. Enzyme Linked Immunosorbent Assays

Enzyme linked immunosorbent assays (ELISA) were performed on un-concentrated supernatants from mature HiN cultures using a capture antibody kit specific for Aβ42 (WAKO Chemicals) as per manufacturer’s instructions. Detection of Aβ_42_ was determined by colorimetric change as compared to standard curve generation.

### 4.5. Calcium Imaging

For calcium imaging, HiNs were incubated with 5 μM Fura-2AM for 1 h before transfer to the stage of a modified upright Olympus BX51WI microscope and then continuously perfused with oxygenated (95% O_2_-5% CO_2_) artificial cerebrospinal fluid (aCSF) with the following composition (in mM): 125 NaCl, 2.5 KCl, 1.25 KH_2_PO_4_, 10 dextrose, 25 NaHCO_3_, 2 CaCl_2_, and 1.2 MgSO_4_, pH 7.4 at room temperature. The microscope was coupled to a xenon light source (Excelitas Technologies, Fremont, CA, USA) and DG5 Sutter filter switch, and Fura-2 fluorescent cellular responses were captured and analyzed using Imaging Workbench Software. Intracellular calcium signals, and RyR-evoked calcium release was assessed by bath application of caffeine (10 mM) for 3 min. A water-soluble nanocrystal formulation of dantrolene (Ryanodex; 10 μM; Eagle Pharmaceuticals, Woodcliff Lake, NJ, USA) was dissolved in aCSF and used as a negative allosteric modulator of the RyR. Fura-2AM responses are reported as peak 340/380 ratio in response to caffeine application after background subtraction.

### 4.6. Statistics

Statistical analysis was performed using Graph Pad Prism 7 and SigmaPlot 12 software. Data are represented as mean ± SEM. Both unpaired t-tests and one-way ANOVA with Tukey’s post hoc analysis were performed where appropriate. Statistical significance was set at *p* < 0.05.

## 5. Conclusions

Taken together, these results demonstrate the power of reprogramming to model human disease in native cell types. Never before have we had the ability to address human disease pathology in human neuronal tissue, and this work confirms a link between AD-related PS1 mutations, amyloid production, tau hyperphosphorylation, calcium dyshomeostasis, and inflammation. As this field evolves, it is feasible to incorporate additional key features from the individuals who provided fibroblast samples, and examine relationships between HiN function and pathology, and higher order functions such as memory or behavioral assays, PET and MRI data of amyloid and tau pathology, functional and structural MRI data, and other health and cognitive assessments. If streamlined, it may even be possible to create personalized therapeutic strategies based on HiN phentoypes and use one’s own ‘neurons’ to screen for effective treatments. Additionally, with the growing sophistication of genomic and proteomic screens and bioinformatics, assessments in the HiN may reveal previously undetected genetic risk factors and identify early pathogenic processes in neurons, and perhaps other brain cells affected in AD, such as microglia and astrocytes. Thus, the findings presented here can serve as a foundation to subsequent studies which can address additional key aspects of AD pathology and validate novel therapeutic strategies.

## Supplementary Materials

The following are available online at https://www.mdpi.com/1422-0067/21/3/1030/s1.
