# Supplementary material for: Human-Induced Neurons from Presenilin 1 Mutant Patients Model Aspects of Alzheimer’s Disease Pathology
Source: Int J Mol Sci. 2020 Feb 4;21(3):1030. doi: 10.3390/ijms21031030 (PMC7037274; doi:10.3390/ijms21031030)
Supplement: Supplementary file 1 [file ijms-21-01030-s001.pdf]

Mutation		Patient ID	iPSC Clone	Oct4 Sox2	TRA_1-60	TRA_1-81	iN	MAP2	NeuN	RyR2
	Control	AG09429	17	+	+	+	+	+	+	+
			37	+	-	+	+	+	+	+
			38	+	+	+	+	+	+	+
		AG02262	46	+	+	+	+	+	+	+
		CW60109	67	+	+	+	+	+	+	+
A246E	AD	AG08711	11	+	+	+	+	+	+	+
			25	+	+	+	+	+	+	+
			26	+	+	+	+	+	+	+
A246E		AG08170	49	+	+	+	+	+	+	+
M146L		AG08446	60	+	+	+	+	+	+	+

Supplemental Table 1: Generated iPSCs and HiN display mature neuronal markers.

Conversion from fibroblast to iPSC was confirmed by the emergence of the expression of OCT-4, SOX2 as measured by qRT-PCR and TRA 1-60 and TRA-181 by IFA. Conversion of iPSC to human induced neurons was confirmed through a combination of qRT-PCR and IFA for the expression of mature neuronal markers of MAP2, NeuN, and RyR2. The ten HiN lines used for this project are shown, with 5 lines from non-AD and 5 lines from PS1 mutant AD

Patient ID	Sex	Age	AD Mutation
AG9429	F	25	N/A
AG02262	M	62	N/A
CW60109	M	38	N/A
AG08711	F	34	A246E
AG08170	M	56	A246E
AG08446	M	38	M146L

Supplemental Table 2: Patient Material Information.

5 fibroblast samples (AG9429, AG02262, AG08711, AG08270, AG08446) and 1 iPSC sample (CW60109) were obtained from the Coriell Institute for Medical Research. Age, sex, and AD mutation are displayed where applicable.
